# Protein expression profiling of inflammatory mediators in human temporal lobe epilepsy reveals co-activation of multiple chemokines and cytokines

**DOI:** 10.1186/1742-2094-9-207

**Published:** 2012-08-30

**Authors:** Anne A Kan, Wilco de Jager, Marina de Wit, Cobi Heijnen, Mirjam van Zuiden, Cyrill Ferrier, Peter van Rijen, Peter Gosselaar, Ellen Hessel, Onno van Nieuwenhuizen, Pierre N E de Graan

**Affiliations:** 1 Department of Neuroscience and Pharmacology, Rudolf Magnus Institute of Neuroscience, Universiteitsweg 100, 3584 CG, Utrecht, The Netherlands; 2 Department of Pediatric Immunology, University Medical Center Utrecht, Heidelberglaan 100, Utrecht, The Netherlands; 3 Department of Neurology and Neurosurgery, University Medical Center Utrecht, Heidelberglaan 100, Utrecht, The Netherlands; 4 Department of Child Neurology, University Medical Center Utrecht, Heidelberglaan 100, Utrecht, The Netherlands; 5 Laboratory of Neuroimmunology and Developmental Origins of Disease, University Medical Center Utrecht, Heidelberglaan 100, 3584 CX, Utrecht, The Netherlands

**Keywords:** Temporal lobe epilepsy, Immune system, Network analysis

## Abstract

Mesial temporal lobe epilepsy (mTLE) is a chronic and often treatment-refractory brain disorder characterized by recurrent seizures originating from the hippocampus. The pathogenic mechanisms underlying mTLE remain largely unknown. Recent clinical and experimental evidence supports a role of various inflammatory mediators in mTLE. Here, we performed protein expression profiling of 40 inflammatory mediators in surgical resection material from mTLE patients with and without hippocampal sclerosis, and autopsy controls using a multiplex bead-based immunoassay. In mTLE patients we identified 21 upregulated inflammatory mediators, including 10 cytokines and 7 chemokines. Many of these upregulated mediators have not previously been implicated in mTLE (for example, CCL22, IL-7 and IL-25). Comparing the three patient groups, two main hippocampal expression patterns could be distinguished, pattern I (for example, IL-10 and IL-25) showing increased expression in mTLE + HS patients compared to mTLE-HS and controls, and pattern II (for example, CCL4 and IL-7) showing increased expression in both mTLE groups compared to controls. Upregulation of a subset of inflammatory mediators (for example, IL-25 and IL-7) could not only be detected in the hippocampus of mTLE patients, but also in the neocortex. Principle component analysis was used to cluster the inflammatory mediators into several components. Follow-up analyses of the identified components revealed that the three patient groups could be discriminated based on their unique expression profiles. Immunocytochemistry showed that IL-25 IR (pattern I) and CCL4 IR (pattern II) were localized in astrocytes and microglia, whereas IL-25 IR was also detected in neurons. Our data shows co-activation of multiple inflammatory mediators in hippocampus and neocortex of mTLE patients, indicating activation of multiple pro- and anti-epileptogenic immune pathways in this disease.

## Background

Epilepsy is a common brain disorder that affects approximately 50 million people worldwide [1]. Mesial temporal lobe epilepsy (mTLE) is a type of epilepsy where seizure activity originates from the hippocampal structures, and is the most common type of partial epilepsy [2]. Approximately 30% of mTLE patients experience seizures that do not respond to treatment with current anti-epileptic medication [3,4]. A common hallmark of mTLE is hippocampal sclerosis; it is characterized by hippocampal neuronal loss combined with gliosis and aberrant axonal sprouting [5,6]. To develop new treatment strategies for drug-refractory patients, there is an urgent need to unravel the molecular mechanisms of epileptogenesis, the complex cascade of molecular, cellular and network changes leading to mTLE [7]. A rapidly growing body of clinical and experimental evidence supports the involvement of the immune system in seizure generation and epileptogenesis [8,9]. For instance, genome-wide mRNA expression profiling in tissue resected from mTLE patients revealed upregulation of multiple genes coding for inflammatory mediators [10,11]. The inflammatory mediators which have been most extensively studied in mTLE are members of the cytokine and chemotactic cytokine (chemokine) families.

Cytokines are soluble polypeptides that play a crucial role in mediating the immune response in both the periphery and the central nervous system (CNS). Prototypical proinflammatory cytokines, such as IL-6, TNFα and the IL-1 family, have been extensively studied in the CNS and are implicated in human mTLE [10,12-14]. Rodent studies have provided evidence for a pro-epileptogenic role of these proinflammatory cytokines (reviewed in [9,15,16]). Tissue resected from mTLE patients also contains increased levels of chemokine transcripts, a specific class of cytokines that provides chemoattractant cues for immune-competent cells such as microglia and leukocytes [17]. Upregulated chemokine transcripts include CCL2, CCL3 and CCL4 (Chemokine (C-C motif) ligand 2 to 4) [10,11], which are considered to be proinflammatory markers in the brain [9,18].

As the inflammatory mediators mentioned above are considered to be pro-epileptogenic, it is expected that interference with inflammatory pathways would ameliorate epileptogenesis and mTLE. However, treatment strategies employing general or specific anti-inflammatory agents in animal models for mTLE have not been fully effective thus far [19-25]. The limited success of anti-inflammatory treatment could be due to the fact that the key targets in epileptogenesis have not yet been identified. Alternatively, the treatment may not only affect proinflammatory pathways, but may concomitantly inhibit beneficial anti-inflammatory pathways. A third possibility is that specific targeting of only one component in the complex pathways of the innate immune response is not sufficient to affect epileptogenesis [26-28].

To address these issues it is essential to map changes in expression of multiple immune mediators in human mTLE tissue. mRNA expression profiling studies have provided the first insight into the regulation of immunological networks in mTLE [10,11]. However, these studies need verification at the protein level, because it is not known whether changes in mRNA expression translate into protein differences. Moreover, recent evidence indicates that large scale post-transcriptional regulation plays a role in mTLE [29,30].

In the present study, we performed protein expression profiling of multiple immune mediators in tissue resected from mTLE patients. We analyzed 40 proteins in each tissue sample using a bead-based multiplex immunoassay (MIA) and compared protein expression levels in homogenates of mTLE patients with hippocampal sclerosis (HS, mTLE + HS), (mTLE-HS) and of non-epileptic autopsy controls. We identified 21 differentially expressed inflammatory mediators, predominantly cytokines and chemokines. Principal component analysis clustered the inflammatory mediators into several components, and follow-up analysis revealed that patient groups could be discriminated based on their unique expression profiles. Our data indicates the upregulation of multiple immune pathways in mTLE and provides new targets for the development of immunological intervention strategies.

## Materials and methods

### Patient selection and tissue collection

Neocortical (medial temporal gyrus) (CX) and hippocampal (HC) tissue samples of pharmaco-resistant mTLE patients were obtained after surgery at the University Medical Centre Utrecht. Patients were selected for surgery according to the criteria of the Dutch Epilepsy Surgery Program [31]. The excision was based on clinical evaluations, interictal and ictal electroencephalography (EEG) studies, magnetic resonance imaging (MRI) and intraoperative electrocorticography (iEcOG). Informed consent was obtained from all patients and all procedures were approved by the Institutional Review Board. Immediately after resection, the tissue was cooled in physiological saline (4°C) and cut on a precooled plate. The neocortex sample was cut in half perpendicular to the surface of the gyrus, the hippocampus into three slices perpendicular to its longitudinal axis. One half of the neocortex sample and the two outer parts of the hippocampus were used for clinical pathological analysis. The remaining neocortical and hippocampal tissue samples were once more divided into a part that was immediately frozen on powdered dry ice and a part that was immersion-fixed in 4% paraformaldehyde in 0.1 M phosphate buffer for 24 h at 4°C. Following fixation, tissue was embedded in paraffin and stored at 4°C. Frozen samples were stored at −80°C. Frozen and paraffin-embedded control brain tissue samples were obtained from non-epileptic post mortem cases without hippocampal aberrations from the Netherlands Brain Bank (NBB no. 618, http://www.brainbank.nl). All control material was collected from donors with written informed consent for brain autopsy and the use of the material and clinical information for research purposes. Only patient samples obtained after a relatively short post mortem delay (range 4 to 20.5 h; mean 6.8 h) and with a pH value close to 6.5 (range 6.36 to 6.88; mean 6.68) were used. Detailed histological examination of the brain material from all patients used in this study showed that all samples were devoid of tumor tissue. Neocortical samples that displayed cortical dysplasia or any other abnormalities that might trigger an immunological response were excluded from the study [32,33]. The quality of all tissue samples was further analyzed in a parallel study [30], showing that after RNA isolation all samples had RNA integrity values (RIN) >6 (range 6.4 to 8.4; mean 7.2) and RIN values did not significantly differ between autopsy control and mTLE patient samples. Table 1 provides a summary of the clinical data of all patients included in the study. Cortical and hippocampal specimens were divided into three groups: a non-epileptic autopsy control group (control, n = 10), a group of mTLE patients without signs of hippocampal sclerosis (mTLE-HS, n = 10) and an mTLE group with hippocampal sclerosis (mTLE + HS, n = 10). The severity of HS was determined by a neuropathologist using the Wyler classification method [34] defining W0 as hippocampal tissue without HS and W4 as tissue with the most severe type of HS. Wyler classification was independently verified on sections of paraffin-embedded tissue.

**Table 1:** Clinical data of mTLE and Control patients

**Patient group**	**Age**	**Sex**	**Pathology/COD**	**Tissue used**	**Seizure frequency**	**iEcOG spikes**	**Anti-epileptic drugs**	**Engel score (1 yr)**
**1) Control**	73	F	subdural hematoma	HC	NA	NA	NA	NA
**2) Control**	58	M	unknown. ALS patient	HC & CX	NA	NA	NA	NA
**3) Control**	62	M	unknown, non-demented control	HC & CX	NA	NA	NA	NA
**4) Control**	94	F	CVA	HC & CX	NA	NA	NA	NA
**5) Control**	71	M	pancreas carcinoma	HC & CX	NA	NA	NA	NA
**6) Control**	64	F	respiratory failure	HC & CX	NA	NA	NA	NA
**7) Control**	70	M	sepsis with broncopneumonia	HC	NA	NA	NA	NA
**8) Control**	50	F	metastasized broncocarcinoma	HC & CX	NA	NA	NA	NA
**9) Control**	48	M	DMT I induced organ failure	HC	NA	NA	NA	NA
**10) Control**	74	M	pulmonary carcinoma	HC & CX	NA	NA	NA	NA
**11) Control**	62	F	renal carcinoma (euthanasia)	CX	NA	NA	NA	NA
**12) Control**	93	F	heart failure	CX	NA	NA	NA	NA
**13) Control**	92	F	cachexia/dehydration	CX	NA	NA	NA	NA
**14) non-HS**	45	M	W0, FCD type1 to 2A in cortex	HC	6	CX	LTG, PHT	1A
**15) non-HS**	46	F	W0, MCD type 1 in cortex	HC	12	CX	CBZ, VPA	1A
**16) non-HS**	46	M	W0, epilepsy after head trauma	HC	90	HC & CX	CBZ, VPA, TPR	1B
**17) non-HS**	42	F	W0, DNT WHO grade I	HC & CX	1	CX	CBZ, LTG, LEV	1A
**18) non-HS**	34	F	W0, cortical cavernoma	HC	1.5	HC & CX	CBZ	1A
**19) non-HS**	40	F	W0, MCD type 1 in cortex	HC	8 ( C )	HC & CX	LEV, LTG, CBZ	1A
**20) non-HS**	43	F	W0, therapy resistant epilepsy	HC & CX	40 ( C )	HC & CX	PHT, LTG	1A
**21) non-HS**	47	M	W0, therapy resistant epilepsy	HC	30	HC	CBZ, VPA, LTG, LEV	3A
**22) non-HS**	28	M	W0, MCD type 1 in cortex	HC	60	HC & CX	CBZ, TPR.	1A
**23) non-HS**	54	M	W0, ganglioglioma WHO grade I	HC & CX	1.5	HC & CX	OXC, LTG, CLO	1A
**24) non-HS**	30	M	W0, therapy resistant epilepsy	CX	8	HC & CX	OXC, LEV, CLO	1A
**25) non-HS**	37	F	W0, therapy resistant epilepsy	CX	1	not measured	LTG, CLO	1A
**26) non-HS**	61	M	W0, therapy resistant epilepsy	CX	5.5	HC & CX	PHT, CBZ	2A
**27) non-HS**	19	M	W0, ganglioglioma WHO grade II	CX	1	None	VPA, LEV	1A
**28) non-HS**	27	F	W0, therapy resistant epilepsy	CX	16	HC & CX	LEV	1A
**29) non-HS**	46	M	W0, cavernoma in uncus	CX	60	HC	CBZ, VPA, OXC, LEV, LTG	1A
**30) non-HS**	44	M	W0, therapy resistant epilepsy	CX	18	HC & CX	TPR, OXC	1A
**31) HS**	41	M	MTS W4	HC & CX	12	not measured	PHT, CLO, CBZ, LTG	1A
**32) HS**	44	F	MTS W2	HC	8 ( C )	not measured	CBZ, OXC, CLO	1A
**33) HS**	41	M	MTS W4	HC	3	not measured	CBZ	1A
**34) HS**	52	F	MTS W4	HC & CX	10	not measured	CBZ, CLO, DZP	2D
**35) HS**	50	M	MTS W4	HC	18	not measured	CBZ, GBP	2A
**36) HS**	36	F	MTS W4	HC & CX	2	not measured	OXC, LZP	no info
**37) HS**	42	M	MTS W4	HC & CX	2	not measured	LEV, LTG	2A
**38) HS**	36	M	MTS W4	HC & CX	10	HC & CX	OXC, PGB	1B
**39) HS**	49	F	MTS W4	HC	8 ( C )	not measured	OXC, CLO	1A
**40) HS**	42	F	MTS W4	HC	4.5	not measured	LEV, LTG, PBT	1A
**41) HS**	36	M	MTS W4	CX	5	not measured	PGB, LTG	1A
**42) HS**	34	F	MTS W4	CX	10 ( C )	HC & CX	LTG, CBZ, VPA,CLO	2A
**43) HS**	49	M	MTS W3	CX	1	None	LTG, CBZ, CLO	1A
**44) HS**	45	M	MTS W4	CX	0.5	not measured	GBP, LTG	1A
**45) HS**	48	M	MTS W4	CX	3.5	not measured	CBZ, LTG, VPA	1A

Hippocampal (HC) and neocortical (CX) homogenates were prepared from tissue of the listed patients as indicated in column 5. All epilepsy patients suffered therapy resistant epilepsy, any additional pathologies are listed in column 4. Seizure frequencies are listed as an average of monthly activity, patients who suffered clustered attacks are marked (C). iEcOG spikes indicate epileptic spike activity detected by iEcOG at surgery. Engel scores were recorded 1 year after surgery [35]. Abbreviations: *ALS* amyotrophic lateral sclerosis, *CBZ* Carbamazepine, *COD* cause of death, *CLO* Clobazam, *CVA* cerebrovascular accident, *CX* temporal neocortex, *DMT I* diabetes mellitus type I, *DNT* Dysembryonic neuroepithelial tumor, *DZP* diazepam, *FCD* focal cortical dysplasia, *GBP* Gabapentin, *HC* hippocampus, *HS* hippocampal sclerosis, *iEcOG* intraoperative electrocorticography, *LEV* Levetiracetam, *LTG* Lamotrigine, *LZP* Lorazepam, *MCD* malformation of cortical development, *MTS* mesial temporal sclerosis, *NA* not applicable, *OXC* Oxcarbazepine, *PBT* Phenobarbital, *PGB* Pregabaline, *PHT* Phenytoin, *PMD* post mortem delay, *RIN* RNA integrity number, *TPR* Topiramate; *VPA* Valproinic acid, *W0 till 4* Wyler score, *WHO grade* world health organization grading scale of malignancy.

### Multiplex bead-based immunoassay analysis

Nissl-stained cryosections were generated to ensure that neocortical samples contained 50% gray and 50% white matter, and that in hippocampal samples all anatomical subregions were equally represented. Subsequently 25 μm cryosections were cut until approximately 20 mg of tissue was collected. This material was stored at −80°C until all samples were collected. The frozen slices were homogenized at 4°C in 400 μl lysis buffer (Lysis M, Roche, Basel, Switzerland), sonicated, centrifuged and passed through a filtering column (0.22 μm, Spin-X, Costar, Sigma-Aldrich, St Louis, MO, USA), frozen and stored (final concentration 0.5ug/ul) at −80°C [36].

Concentrations of 39 immune modulators (listed in Table 2) were measured using a multiplex bead-based immunoassay (MIA) as previously described. [37]. Capture and detection antibody pairs and recombinant proteins used for the standard curves were purchased from commercial sources as described previously [38] (and Table 3). Carboxylated polystyrene microspheres were purchased from Bio-Rad Laboratories (Hercules, CA, USA). Covalent coupling of the capture antibodies to the microspheres was performed as previously described [37,38]. Optimal working conditions and sample dilutions were determined for each antibody and calibration curves from recombinant protein standards were prepared using two-fold dilution steps in Lysis M buffer used for homogenization [38]. All assays were carried out directly in a 96 well 1.2 μm filter plate (Millipore, Billerica, MA, USA) at room temperature and protected from light. A mixture containing 1000 microspheres per antigen (total volume 10 μl/well) was incubated together with a standard, homogenate or lysis buffer for 1 h at room temperature. Next, 10 μl of a cocktail of biotinylated antibodies (16.5 μg/ml each) was added to each well and incubated for an additional 60 min Beads were then washed with phosphate buffered saline (PBS) supplemented with 1% bovine serum albumin (BSA) and 0.5% Tween 20 at pH of 7.4. After incubation for 10 min with 50 ng/well streptavidin R-phycoerythrin (BD Biosciences, San Diego CA, USA) and washing twice with PBS-1% BSA-0.5%-Tween 20 pH 7.4. Fluorescence intensity of the beads was measured in a final volume of 100 μl HPE buffer (Sanquin Reagents, Amsterdam, The Netherlands) and buffer values were subtracted from all readings. Measurements and data analysis were performed using the Bio-Plex system in combination with the Bio-Plex Manager software version 4.1 (Bio-Rad Laboratories). CCL4 levels were measured separately using a fluorokine kit (R&D Systems, Abingdon, UK) according to the manufacturer’s instructions.

**Table 2:** Protein levels inflammatory mediators

**Protein**	**Hippocampal expression (pg/ml)**						**Cortical expression (pg/ml)**						**P-value**
**Chemokines**	***Cntrl***		***mTLE-HS***		***mTLE + HS***		***Cntrl***		***mTLE-HS***		***mTLE + HS***		
**CCL22/MDC**	**0.3**	(0–0.7)	**0.8**	(0.4-1.6)	**0.9**	(0.3-1.4)	**0.2**	(0–0.6)	**0.6**	(0.3-1.2)	**0.9**	(0.7-1.3)	**1.74E-08**
**CCL5/RANTES**	**8.1**	(4–15.8)	**77.4**	(40.9-238.1)	**49.4**	(18.5-167.7)	**10.9**	(1.6-18.5)	**26.0**	(18.5-122.3)	**31.5**	(17.4-38.5)	**1.60E-08**
**CCL4/MIP1β**	**0**	(0–0)	**19.4**	(4–122.3)	**18.9**	(6.2-95.3)	**0**	(0–0)	**0**	(0–7.6)	**0.0**	(0–33.6)	**2.11E-08**
**CXCL9/MIG**	**3.2**	(1.1-11)	**9.0**	(6.7-16.7)	**11.0**	(4.4-17.1)	**5.7**	(2.4-7.7)	**8.3**	(5.7-12)	**10.7**	(7.0-12.3)	**5.99E-07**
**CCL2/MCP1**	**1.1**	(0–4.3)	**11.4**	(2.1-52.8)	**9.3**	(2.1-29.0)	**0.0**	(0–2.9)	**0.7**	(0–4.6)	**0.3**	(0–18.2)	**4.13E-06**
**CCL3/MIP1α**	**44.4**	(11.2-106.2)	**94.5**	(62–177.4)	**94.6**	(62–317.1)	**35.6**	(20.1-57.4)	**62.0**	(35.6-104.9)	**85.1**	(59.0-97.2)	**9.13E-05**
**CCL19/MIP3β**	**0**	(0–0)	**0.5**	(0–1.6)	**0**	(0–1)	**0**	(0–0)	**0**	(0–1.7)	**0.4**	(0–1.3)	**9.90E-05**
CCL18/PARC	16.6	(0.2-151.6)	24.2	(1.8-177.4)	14.0	(0–29.5)	13.7	(0–132.7)	3.3	(0–31.9)	0.7	(0–37.6)	0.001966
CXCL8/IL-8	0.8	(0–13.8)	2.7	(0–26)	1.0	(0.3-20.1)	0.6	(0–1.6)	0.5	(0–10.3)	0.9	(0–3.6)	0.02352
CCL11/Eotaxin	0		0		0		0		0		0		b.d.
CCL17/Tarc	0		0		0		0		0		0		b.d.
CXCL10/IP-10	0		0		0		0		0		0		b.d.
**Cytokines**													
**IL-7**	**0**	(0–5.6)	**8.3**	(3.8-9.6)	**7.3**	(0.6-12.2)	**0.9**	(0.0-4.6)	**8.7**	(4.6-14.5)	**11.0**	(8–12.7)	**5.07E-15**
**IL-13**	**2.6**	(0.9-6.8)	**13.4**	7.9-20.5)	**11.2**	(8.1-17.4)	**6.4**	(2.6-12.5)	**12.7**	(10.3-17.4)	**15.4**	(10.7-16.5)	**7.60E-13**
**IL-22**	**92.5**	(58.3-212.2)	**351.2**	(236.6-540.6)	**304.1**	(244.8-449.9)	**212.2**	(122.8-359.4)	**380.0**	(293.8-548.8)	**425.2**	(261.1-499.3)	**1.65E-12**
**IL-5**	**0**	(0–1.2)	**2.8**	(1.5-7.4)	**2.8**	(0.3-5.5)	**0**	(0–1)	**2.3**	(1–6.7)	**5.0**	(2–6.1)	**2.06E-07**
**IL-25/IL-17E**	**726.9**	(273.4-1780)	**868.3**	(348.3-1616)	**1976.4**	(258.5-2499)	**303.3**	(109.9-757.4)	**673.5**	(483.8-1234.5)	**922.1**	(468.7-1327.5)	**1.93E-05**
**IL-1RA**	**13.5**	(6.7-26.8)	**19.0**	(13.5-33.4)	**20.7**	(12.3-29)	**15.7**	(13.5-31.2)	**29.0**	(13.5-165.2)	**53.1**	(26.8-92.2)	**2.96E-05**
**IL-1a**	**0**	(0–0.2)	**0.3**	(0–3)	**0.3**	(0–1.3)	**0**	(0–0)	**0**	(0–0.9)	**0.3**	(0–1.6)	**5.06E-05**
**IL-27**	**40.9**	(0–153.8)	**33.7**	(0–133.4)	**132.1**	(0–370.5)	**0**	(0–42.3)	**28.6**	(0–89.7)	**49.4**	(29.4-78.7)	**8.93E-05**
**IL-10**	**2.2**	(0–5.9)	**1.4**	(0–3.4)	**7.8**	0-12.4)	**0**	(0–2.2)	**0.5**	(0–1.9)	**0.9**	(0–3.4)	**1.02E-04**
**IFNα**	**22.5**	(12.8-41.5)	**31.5**	(22.7-58.5)	**66.3**	(10.1-114.3)	**27.0**	(4.1-73.1)	**41.3**	(26.9-51.5)	**38.5**	(30.7-59)	**3.40E-04**
MIF	11135.4	(7054–13156)	8200.7	(6406–19843)	6907.3	(4910–9650)	11062.2	(4645–17980)	7112.0	(4645–9872)	7180.8	(5547–9470)	0.0024
IL-1β	0.2	(0–0.9)	0.8	(0.2-1.7)	0.9	(0–1.7)	0.2	(0–0.9)	0.5	(0–1.5)	0.9	(0–1.7)	0.0063
IL-23	298.9	(190.4-568.7)	575.1	(473.2-675)	556.2	(311.2-691)	308.1	(238.2-717)	500.2	(286.7-633)	543.2	(372.9-710)	0.0063
IL-4	0	(0–0.1)	0.1	(0–0.2)	0	(0–0.2)	0	(0–0)	0	(0–0.2)	0.1	(0–0.2)	0.0234
IL-6	0	(0–11.7)	0	(0–3.9)	0	(0–1.1)	0	(0–2.1)	0	(0–0)	0.0	(0–0)	0.0428
IL-21	336.5	(117.7-944.2)	510.6	(0–944.2)	831.0	(55.8-1270.4)	431.2	(0–1009.1)	415.4	(0–847.1)	654.1	(179.9-1534)	0.0848
TNFα	10.4	(8.6-12.7)	11.5	(7.0-13.2)	11.2	(8.2-13.2)	9.3	(6.3-12.9)	10.6	(6.9-12.3)	10.5	(9.2-13.2)	0.1341
IL-18	3.7	(1.7-14.2)	3.2	(1.5-5.2)	2.1	(1.1-23.8)	5.0	(1.6-163.1)	2.9	(1.7-12.4)	2.9	(2.2-4.8)	0.1481
IL-6R	1.4	(0–10)	1.4	(0–5.6)	4.5	(1.4-8.9)	3.4	(0–17)	3.4	(0–10)	4.5	(0–7.8)	0.4944
IL-2	0		0		0		0		0		0.0		b.d.
IL-12p70	0		0		0		0		0		0.0		b.d.
**Other**													
**VCAM1**	**219.7**	(33.9-828.3)	**985.5**	(548–1722.6)	**1181.3**	(364.5-1649)	**210.3**	67.3-527.2)	**817.5**	(444.9-1287)	**1239.9**	(740.4-1551)	**2.71E-11**
**VEGF**	**10.0**	(0.4-28.8)	**30.3**	(18.6-47)	**31.1**	(7.2-59.3)	**4.5**	(0–12.8)	**22.9**	(12.8-39.3)	**32.6**	(17.1-36.3)	**3.70E-10**
**HGF**	**33.0**	(26.7-54.5)	**105.2**	(65.8-157.4)	**90.3**	(34.6-156.4)	**24.0**	(19–50.3)	**71.3**	(37–207.7)	**52.4**	(32.2-83.6)	**1.38E-07**
**ICAM1**	**9099.6**	(3364–17901)	**5400.2**	(3174–9985)	**16011.1**	(5781–26620)	**5039.5**	(1532–16739)	**3440.4**	(1532–10754)	**4698.9**	(1861–8725)	**9.13E-05**
Cathepsin S	463.3	(284.2-1010)	311.9	(129.1-525.7)	218.0	(160.4-425.5)	350.6	(198.2-993.6)	303.8	(198.2-478.7)	327.2	(190.4-413)	0.0025
TIMP-1	101.0	(36.2-2243.7)	124.3	(81.6-382.5)	103.2	(50.7-204)	81.6	(53.7-137.7)	97.7	(64.5-324.4)	112.5	(83.5-305.6)	0.2672
Adiponectin	415.0	(14–1412.5)	727.8	(193.3-1643)	480.3	(221–1787.4)	722.4	(22.4-1979.9)	490.0	(22.4-940.7)	349.3	(0–1751)	0.4253

MIA was performed on selected chemokines, cytokines, growth factors and adhesion molecules. HC and CX protein levels were determined in controls and 2 mTLE patient groups (− and + HS).Inflammatory proteins are listed in order of **overall** significance (**ANOVA/Kruskal-Wallis**). Proteins in bold show significant changes between groups (ANOVA/Kruskal-Wallis, p < 0.0015). Levels are presented as medians and range (between brackets). Abbreviations: *b.d*. below detection, *HGF* hepatocyte growth factor, *ICAM1* intercellular cell adhesion molecule 1, *IFNα* interferon type 1α, *IP10* Interferon gamma-induced protein 10, *MCP1* macrophage derived chemokine, *MDC* macrophage derived chemokine, *MIF* Macrophage migration inhibitory factor, *MIG* monokine induced by interferon γ, *MIP1α* Macrophage inflammatory protein-1α, *MIP1β* Macrophage inflammatory protein-1β, *MIP3β* Macrophage inflammatory protein-3β, *PARC* pulmonary and activation-regulated chemokine, *RANTES* Regulated upon activation normal T cell express sequence, *TARC* thymus and activation regulated chemokine, *TIMP-1* tissue inhibitor of metalloproteinases, *TNFα* tumor necrosis factor, *VCAM1* vascular cell adhesion molecule 1, *VEGF* vascular endothelial growth factor.

**Table 3:** Recombinant proteins and antibody sets used in MIA

**Antigen**	**Protein source**	**Antibody set**
CCL4	R&D	R&D
CCL19	R&D	R&D
IL-1RA	R&D	Bioledgend
IL-6R	R&D	Sanquin
IL-7	BD	BD
IL-21	Abnova	eBioscience
IL-22	Peprotech	Peprotech
IL-23	R&D	eBioscience
IL-25	R&D	R&D
IL-27	R&D	R&D
IFNa	eBioscience	eBioscience
VEGF	R&D	R&D
HGF	R&D	R&D
Cathepsin S	R&D	R&D
TIMP1	R&D	R&D
Adiponectin	R&D	R&D

R&D systems, UK; Bioledgend, USA; Sanquin, The Netherlands; Abnova, Taiwan; Peprotech, USA; eBioscience, USA.

MIA was performed on selected chemokines, cytokines, growth factors and adhesion molecules. HC and CX protein levels were determined in controls and two mTLE patient groups (− and + HS). Inflammatory proteins are listed in order of overall significance (ANOVA/Kruskal-Wallis). Proteins in bold show significant changes between groups (ANOVA/Kruskal-Wallis, *P* < 0.0015). Levels are presented as medians and range (between brackets). Abbreviations: b.d., below detection; HGF, hepatocyte growth factor; ICAM1, intercellular cell adhesion molecule 1; IFNα, interferon type 1α; IP10, interferon gamma-induced protein 10; MCP1. monocyte chemotactic protein-1; MDC, macrophage-derived chemokine; MIF, macrophage migration inhibitory factor; MIG, monokine induced by interferon γ; MIP1α , macrophage inflammatory protein-1α; MIP1β, macrophage inflammatory protein-1β; MIP3β, macrophage inflammatory protein-3β; PARC, pulmonary and activation-regulated chemokine; RANTES, regulated upon activation normal T cell express sequence; TARC, thymus- and activation-regulated chemokine; TIMP-1, tissue inhibitor of metalloproteinases; TNFα. tumor necrosis factor; VCAM1, vascular cell adhesion molecule 1; VEGF, vascular endothelial growth factor.

### Immunohistochemistry and immunofluorescence

**Figure 1:**
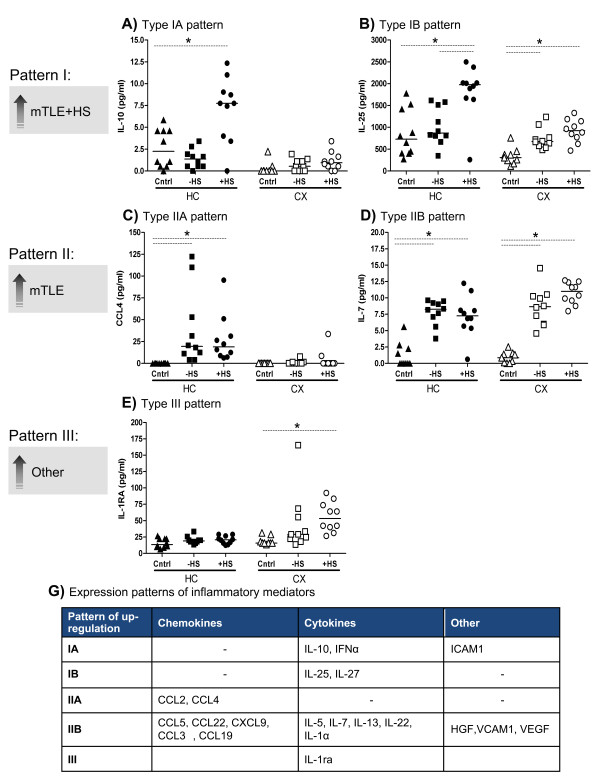
**HC and CX expression patterns of inflammatory proteins in mTLE.** Scatter plots of protein levels per individual patient, per group. Horizontal lines represent the median expression level of the group. Post-hoc analysis of the data revealed three expression patterns among the patient groups. We classified them as Type I to III (I: **A** and **B**, II: **C** and **D**, III: **E**). Additionally, different patterns were detected depending on the type of tissue, hence we classified an up-regulation in HC only as **A** (**A** and **C**) and an up-regulation in both HC and CX as **B** (**B** and **D**). * indicates significant difference. All significantly changed proteins showed one of these three expression patterns (**G**) (see Figure 2)

Hippocampal immunohistochemistry (IHC) was performed on 7 μm paraffin sections of the same patients (n = 6 per group) as used in the MIA (CCL4 polyclonal goat, 1:200, Santa Cruz Biotechnology, Santa Cruz, CA, USA; IL-25 monoclonal mouse, 1:800, R&D systems, Abingdon, UK). We tested several antibodies against immune modulators with pattern 1 and 2 (Figure 1). Based on their superior quality for immunocytochemistry on paraffin section we choose IL-25 and CCL4. Serial antibody dilution curves were prepared in phosphate buffered saline (PBS) containing 0.2%Triton-X100 to determine optimal working concentrations. After rehydration, all sections were subjected to antigen retrieval using microwave treatment (in 0.01 M, Na^+^ Citrate pH 6.0), non-specific binding was blocked by incubating sections with 0.3% H_2_0_2_ for 30 min at room temperature and with fetal calf serum (IL-25) or normal rabbit serum (CCL4) for 30 min at 37°C. Sections were incubated with primary antibodies overnight at 4°C. The secondary antibodies used were biotinylated rabbit anti-goat and biotinylated horse anti-mouse (Dako Cytomation, Glostrup, Denmark). Signals were visualized using the avidin-biotin method (Vectastain ABC Elite kit; Vector Laboratories, Burlingame, CA, USA) with 3,3′-diaminobenzidinetetrachloride (DAB) as the chromogen (Sigma Chemical Co., St. Louis, MO, USA). By visual inspection, sections were ranked according to overall IR levels by two independent observers blinded to the experimental design. No immunostaining was observed when the protocol was completed with primary antibodies preabsorbed with their respective antigens, or without the primary antibodies.

Double immunofluorescence labeling was performed as described for IHC, but without the 0.3% H_2_0_2_ blocking step. Anti-GFAP (polyclonal rabbit, 1:6000 Dako Cytomation, Glostrup, Denmark), Iba1 (polyclonal rabbit, 1:200, WAKO Pure Chemical Industries, Neuss, Germany), Vimentin (monoclonal mouse,1:2400, Dako Cytomation, Glostrup, Denmark) and NeuN (polyclonal rabbit, 1:800, Millipore, Billerica, MA, USA) were used as CNS cell-type markers. Secondary antibodies used were donkey anti-goat Alexa 488 and goat anti-mouse Alexa 488 (Invitrogen, Molecular Probes, Eugene, OR, USA) for CCL4 and IL-25, donkey anti-mouse Alexa 555 and donkey anti-rabbit Alexa 555 (Invitrogen, Molecular Probes, Eugene, OR, USA) for the cell-type markers. Finally, Sudan Black dye was used to reduce autofluorescence [39]. Controls without primary antibody were devoid of fluorescent staining.

### Statistical analyses

To determine overall statistical significance of differences in protein expression between patient groups, data from all detectable inflammatory mediators were analyzed using a one-way ANOVA (if normally distributed) or Kruskal-Wallis test (if not normally distributed). When significant group differences were present (Bonferroni corrected for multiple testing: *P* = <0.0015), additional post hoc tests (Table 4) were performed (Students *t*-test or Mann–Whitney U with Bonferroni correction: *P* = <0.008).

**Table 4:** Summary of all p-values for the post hoc tests

**Parameter**	**HC**	**HC**	**HC-CX**	**HC**	**HC-CX**	**CX**	**CX**
	**+HS vs – HS**	**+HS vs Cntrl**	**+HS vs + HS**	**-HS vs Cntrl**	**-HS vs –HS**	**+HS vs Cntrl**	**-HS vs Cntrl**
IL1 RA	0.64497528	0.116	**0.0008**	0.137	0.108	**0.0005**	0.027
IL1a	0.24252404	**0.0007**	0.687	**0.003**	0.0722	**0.0021**	0.171
IL-5	0.706	**0.0002**	0.0261	**0.001**	0.705	**1.32E-06**	**0.001**
IL-7	0.853	**6.65E-05**	0.007	**3.28E-07**	0.469	**1.78E-10**	**7.37E-06**
IL-10	**4.1619E-06**	**0.004**	**2E-06**	0.138	0.079	0.068	0.305
IL-13	0.25819279	**2.78E-06**	0.0449	**7.35E-07**	0.518	**1.16E-06**	**4.90E-05**
IL-22	0.491	**4.86E-07**	0.0149	**9.13E-07**	0.478	**1.23E-05**	**0.00023**
IL-25	**1.8845E-05**	**0.001**	**1E-07**	0.470	0.0865	**3.81E-05**	**0.001**
CCL2	0.376	**0.0009**	0.0185	**0.0006**	**0.0001**	0.372	0.227
CCL3	0.521	*0.015*	0.106	**0.002**	0.0132	**1.34E-06**	**0.002**
CCL5	0.250	**0.006**	0.0661	**0.001**	0.0166	**5.78E-07**	**0.0002**
CCL19	0.258	0.045	0.203	**0.0015**	0.2107	**0.002**	0.126
CCL22	0.99	**0.00029**	0.5204	**0.0002178**	0.1207	**2.00E-07**	**0.001**
CXCL9	0.168	**0.00020**	0.0958	**0.001**	0.249	**1.70E-05**	**0.003**
HGF	0.327	**0.001**	**0.0021**	**9.61E-08**	0.142	**0.001**	**0.006**
sICAM1	**5.9897E-05**	**0.004**	**2E-05**	0.052	0.281	0.136	0.147
sVCAM1	0.326	**3.01E-05**	0.9371	**7.48E-05**	0.1186	**6.88E-09**	**0.00011**
VEGF	0.714	**0.001**	0.5836	**8.71E-05**	0.0925	**3.09E-10**	**3.07E-05**
IFNa	**0.00177**	**0.002**	*0.014*	0.036	0.1402	0.327	0.318
IL-27	**0.0012**	*0.008*	**0.0051**	0.933	0.4785	**3.62E-06**	0.022
CCL4	0.795	**0.0000108**	0.022	**0.00001**	**0.00004**	0.49	0.27

All significantly different inflammatory mediators (ANOVA/Kruskal-Wallis) were subjected to post-hoc testing (Students-*T* test/Mann–Whitney U). Significant p-values are depicted in bold.

A univariate general linear model analysis was used to assess possible confounding effects of age and gender. Using age or gender as covariate did not result in loss of significance for any of the previously significant group differences (*P* = <0.0015), nor did age and gender significantly predict the outcome.

In the autopsy control data set Pearson’s correlation tests did not reveal significant correlations between protein expression and brain pH or post mortem delay (PMD), thus ruling out pH and PMD as confounders (CC < 0.7 *P* = > 0.0015). Also, using RNA integrity numbers (RIN) measured for a different study of these control samples, no correlation was found between inflammatory mediator levels and RIN values [30]. To assess correlations between expression levels of inflammatory mediators (all proteins with *P* = <0.05) and known clinical parameters (Engel score, seizure frequency, iEcOG spikes), we performed correlation analyses using a Pearson’s correlation (if normally distributed) or a Spearman’s rank test (if not normally distributed).The cut-off for a correlation was set at 0.8 with a Bonferroni corrected *P* value *P* = <0.0017. All positive correlations between inflammatory mediators were plotted to form networks using Cytoscape.

Finally, all data from the detectable inflammatory mediators were clustered into components (factors) using an unbiased principle component analysis (PCA) (see Marengo *et al*. 2010 [40]) (in SPSS also known as factor analysis). As bivariate analyses showed that expression data for multiple proteins were correlated, which is in line with functional relations between cytokines, we chose the oblimin rotation, which allows correlation between factors, rather than the often used varimax rotation, which does not allow correlation between factors. The number of extracted components was based on examination of the scree plot and eigenvalues (>1.0). Interpretation of the components was based on mediators with factor loadings above 0.4 (16% explained variance). When there were missing values (in one patient, for two cytokines) they were replaced with the group mean. Rotations for all data converged within 50 iterations. All inflammatory mediators were used to calculate individual components scores for each participant (using regression to determine the component or factor scores). These were saved and subsequently used to analyze group differences and construct PCA plots.

Programs used for the statistical analyses and graphical representations were SPSS (SPSS 15 for Windows), Graphpad Prism 4 and Cytoscape 2.8.

## Results

### Protein expression profiling of the immune system in human mTLE

**Figure 2:**
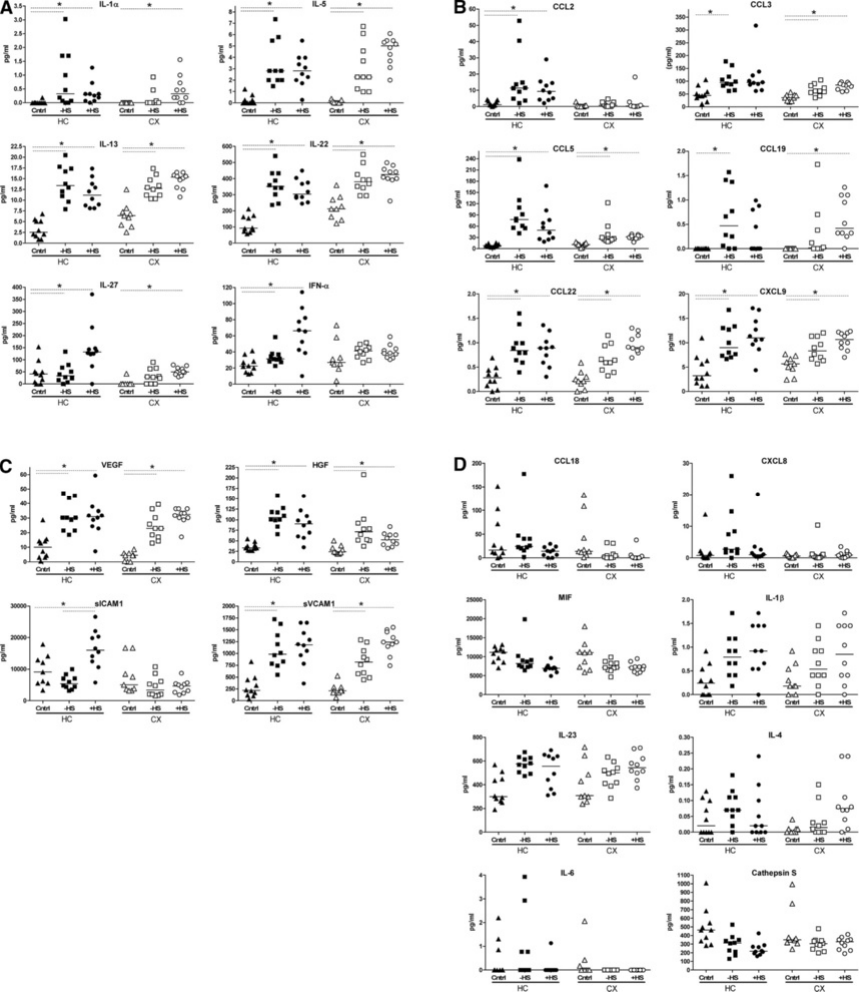
**HC and CX expression patterns of other inflammatory proteins.** Interleukins (**A**), chemokines (**B**), miscellaneous inflammatory proteins (**C**), and inflammatory proteins with p-values 0.0017-0.05 which were not tested post-hoc (**D**). Scatterplots of individual protein levels per patient group. Horizontal lines represent the median expression level of the group

To generate protein expression profiles of inflammatory mediators in individual mTLE patients, we analyzed 40 proteins in each brain sample using a multiplex bead-based immunoassay. To assess mTLE-associated differences in expression profiles in hippocampus (HC) and neocortex (CX), we compared three patient groups, mTLE patients without HS (mTLE-HS), mTLE patients with HS (mTLE + HS), and autopsy controls (for patient details see Table 1). This experimental setup has been previously used by us and others [41-44]. In samples of mTLE patients, we could reliably detect 35 proteins, whereas due to the lower expression levels in autopsy control tissue we could reliably detect 32 proteins. Table 2 summarizes the data set (median and concentration range) of all inflammatory mediators measured in all HC and CX samples in the three patient groups. Statistical analysis revealed that 21 inflammatory mediators showed differential expression across patient groups (Bonferroni-corrected *P* value <0.0015); mediators are ranked according to level of significance. A univariate general linear approach and Pearson’s correlation tests revealed no significant covariate influence or correlations for gender, age, post mortem delay, anti-epileptic drugs (AED) or pH. Post hoc testing showed that all 21 differentially expressed mediators were upregulated in mTLE + HS and/or mTLE-HS tissue compared to autopsy controls. Two inflammatory mediators (MIF, *P* = 0.0024 and Cathepsin S, *P* = 0.0025) showed a tendency to upregulation in autopsy controls compared to both mTLE groups (Table 2 and Figure 2D), but this upregulation did not pass the strict Bonferroni correction.

### Inflammatory mediators show distinct patterns of upregulation across patient groups and tissues

To further analyze the upregulation of individual inflammatory mediators between patient groups, we generated scatter plots per mediator showing the protein concentration in the HC and CX of each individual patient (Figure 1 and Figure 2). These plots clearly showed that upregulation of mediators in HC and CX tissue can be divided into two patterns (classified as pattern I and II). For instance, IL-10 and IL-25 showed upregulation in mTLE + HS tissue compared to mTLE-HS and autopsy controls (pattern I, Figure 1A and 1B), whereas CCL4 and IL-7 showed upregulation in both mTLE groups compared to autopsy controls (pattern II, Figure 1C and 1D). Interestingly, IL-1RA was only upregulated in the CX of mTLE + HS patients (Figure 1E).

Upregulation of some mediators, which we classified type A, was only found in the hippocampus (Figure 1A and 1C), whereas others (classified as type B) showed upregulation in both hippocampal and neocortical tissue (Figure 1B and 1D). A summary of the response types of all significantly upregulated mediators is provided in Figure 1G.

These results show that in both mTLE-HS and mTLE + HS patients multiple components of the immune response are upregulated. Upregulation can be found in the HC and the CX, and inflammatory mediators show distinct patterns of upregulation.

### Cellular localization of IL25 and CCL4

Our next step was to independently verify upregulation and to analyze the distribution and cell types expressing one inflammatory mediator in each of the two major expression patterns. We performed immunohistochemistry (IHC) for IL-25 (pattern I) and CCL4 (pattern II) on hippocampal sections of a subset (n = 6) of patients from each of the three groups also used in the MIA. Visual inspection of overall immunoreactivity (IR) for IL-25 revealed increased expression in all sections from mTLE + HS patients compared to mTLE-HS and autopsy control patients, thus confirming our MIA data (Figure 1B).

**Figure 3:**
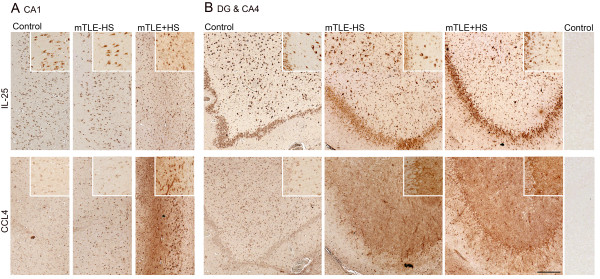
**Hippocampal expression patterns of IL-25 and CCL4 in Control, mTLE-HS and mTLE + HS patients.** Photomicrographs showing typical examples of Il-25 and CCL4 staining in the hippocampal CA1 region (**A**) and the DG/CA4 region (**B**). IL-25 immunoreactivity (IR) is evident in cells with neuronal morphology in all three patient groups (**A**, **B**); note the increased IL-25 staining in the mTLE + HS hippocampus in small cells. CCL4 IR is low in controls (**A**, **B**). Increased CCL4 IR is detected in both mTLE patient groups in the DG-CA4 area (**B**) and in the CA1 area of mTLE + HS patients (**A**). Scale bar = 200 μm.The insets are higher power magnifications taken from the same anatomical area

**Figure 4:**
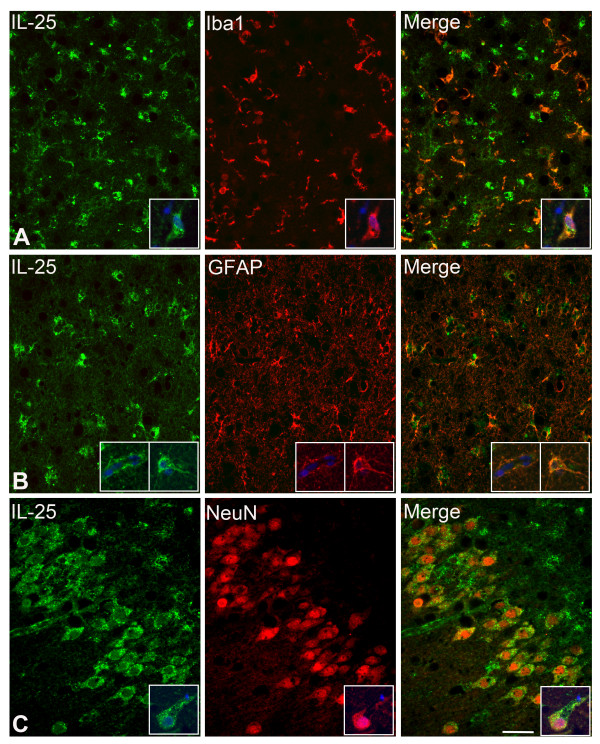
**IL-25 IR in neurons, astrocytes and microglia.** IFC reveals IL-25 IR (in green) co-localizing with three CNS cell type markers (red), (**A**) Iba1, a microglial marker, (**B**) GFAP, an astrocytic marker and (**C**) NeuN, a neuronal marker. **Panel A,B**: CA4; **Panel C**: dentate gyrus. Note that IL-25 IR is also present in the astrocytic endfeet surrounding the bloodvessels (**B**). The insets are higher power magnifications taken from a representative area, with the added nuclear marker DAPI (blue). Scale bar = 40 μm

IL-25 IR was found in all three patient groups in the principle neuronal layers of the hippocampus, the pyramidal layer of the cornu ammonis (CA) (subfields 1 to 4) and the granule cell layer in the dentate gyrus (DG). In mTLE patients the increased IL-25 IR was most apparent in the CA1 (Figure 3A) and the DG-CA4 region (Figure 3B) of the hippocampus. This increase in IL-25 IR was due to labeling of cells with a glial morphology (see also Figure 4A and 4B). As expected, the number of IL-25 IR neurons was decreased in mTLE patients compared to mTLE-HS and controls as a result of neuron loss in the sclerotic hippocampus [5].

Hippocampal CCL4 IR was higher in all sections of both mTLE groups compared to autopsy controls (Figure 3),thus confirming our MIA data (Figure 1C). CCL4 expression in both mTLE groups was most pronounced in the CA1 and the hilar region of the hippocampus (Figure 3).

**Figure 5:**
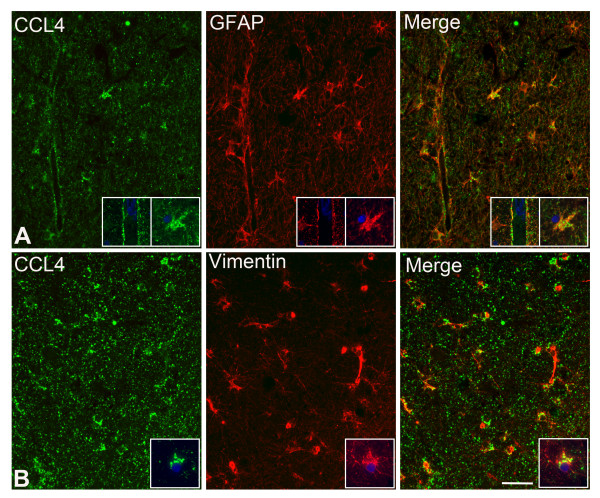
**CCL4 IR in astrocytes and reactive astrocytes.** IFC reveals CCL4 (in green) co-localizing with two types of glial cell markers (red), (**A**) astrocytic marker GFAP and (**B**) reactive astrocytic marker Vimentin. CCL4 IR is also present in the astrocytic endfeet of the GFAP positive astrocytes (**A**). Pictures are taken of representative areas in the CA4 region. The insets are higher power magnifications taken from the same anatomical area, with the added nuclear marker DAPI (blue)**.** Scale bar = 40 μm

To identify the cell types expressing IL-25 and CCL4 IR, we performed double-label immunofluorescence on mTLE + HS patients (Figure 4 and Figure 5). IL-25 IR co-localized with Iba1-positive microglial cells, GFAP-positive astrocytes and NeuN-positive neuronal cells (Figure 4). CCL4 was predominantly detected in Vimentin-positive reactive (Figure 5A) and GFAP-positive astrocytes (Figure 5B). Interestingly, both IL-25 and CCL4 IR were also detected in GFAP-positive astrocytic endfeet surrounding blood vessels (Figure 4B and 5A, small insets). To exclude T cells as prominent source of cytokines we performed IHC for these cells (data not shown). In line with results published by others [12,45], we only detected a small number of T cells in the hippocampus of mTLE patients. Together, our immunocytochemistry data show distinct hippocampal staining patterns for IL-25 and CCL4, particularly in the dentate gyrus, where IL-25 but not CCL4 expression is prominent in the principle neurons in the granule cell layer. In mTLE + HS patients the increased IL-25 and CCL4 expression (compared to controls) appears to involve glia cells, whereas increased IL-25 expression is also found in principle neurons.

### Principle component analyses revealed pathology-associated immunological profiles in mTLE

**Figure 6:**
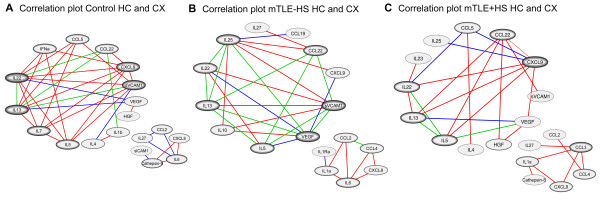
**Correlation network plots for all patient groups.** Network plots depicting significant correlations between levels of inflammatory mediators in Control patients (**A**), mTLE -HS patients (**B**) and mTLE + HS patients (**C**). Line (edge) color indicates in which tissue correlation was found: red = HC, blue = CX, green = HC & CX. Per patient group two main non-overlapping correlation networks were identified. Note that correlation networks are remarkably similar among the patient groups. Inflammatory mediators like IL-5, IL-13 and IL-22 all display >4 correlations with other inflammatory mediators. Only proteins that were in a network of more than 6 correlations (nodes) are depicted. In addition, 5 significant correlations were found in the total data set. In Control HC IL-10 & CCL3 correlated with IL-25 and in mTLE-HS CX Cathepsin-S correlated with ICAM1. All correlations had a correlation coefficient > 0.85 with a Bonferoni corrected p-value < 0.0017

To investigate relationships between various upregulated mediators, we conducted unbiased correlation network and principle component analyses on data from all patients, irrespective of their pathology. First, correlation analyses using Pearson’s correlation showed that many cytokines were co-regulated in patterns that were remarkably similar in the three patient groups and segregated in two main networks (Figure 6). In most cases, patients with high expression of proteins in the first network had lower levels of proteins in the second network, and vice versa (data not shown).

**Figure 7:**
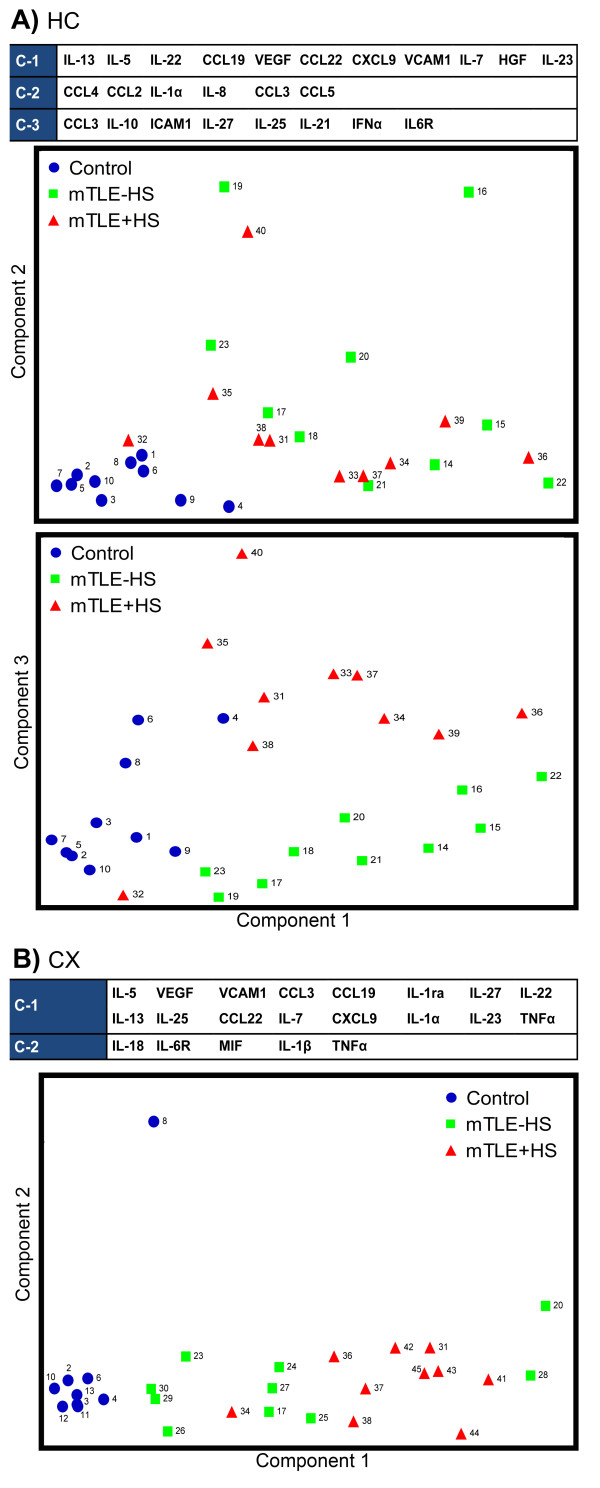
**Major components identified by principle component analyses reveal pathology-specific immunological networks in the HC and the CX.** PCA results for the main components identified by the analysis in the HC (**A**) and the CX (**B**). Proteins are depicted in sequence of factor loading, all depicted proteins of the components have a factor loading of >0.4 (is >16% of explained variance). Individual patient component scores were plotted against each other and revealed patient group-dependent clusters, component 1 versus 2 (**A**) segregated both mTLE patient groups from controls in HC (**A**) and CX (**B**), whereas the PCA plot for component 1 versus 3 in the HC segregated all three patient groups (**B**). C-1 to 3 = component 1 to 3

Principle component analyses (PCA) on all inflammatory mediators above the detection limit (see Table 2), confirmed the correlation analyses and revealed clustering of the proteins in eight principle components. These eight components explained 88.5% (HC) and 84% (CX) of the variance detected in all samples (Table 5A and B). The major components, which explained 63% (HC) and 50.1% (CX) of the variance, are depicted in Figure 7 with their corresponding plots. The first and the third HC components contained all three types of inflammatory mediators we investigated (chemokines, interleukins, growth factors and adhesion molecules), while the second HC component predominantly consisted of chemokines (Figure 7A). Follow-up analysis on the individual factor scores obtained from the PCA, revealed that HC components 1 and 2 differed as whole clusters between both mTLE groups and controls, whereas the HC component 3 differed between mTLE + HS versus mTLE-HS and controls (Table 5A). The PCA plots (Figure 7A, bottom panel) clearly showed that patient groups can be discriminated based on the expression patterns of inflammatory mediators in components 1 and 3. Interestingly, patient 32 of the mTLE + HS group did not cluster with the rest of the mTLE + HS group (Figure 7A). Neuropathological reassessment revealed only mild sclerosis (W2 score) for this patient (Table 1). Component 1 of the CX tissue contained sixteen inflammatory mediators, of which nine overlapped with the HC first component. Follow-up analyses of the components scores obtained from the PCA of the neocortical expression data (Figure 7B) also showed that the three patient groups could be discriminated, yet to a lesser degree, as only CX component 1 differed significantly between patient groups (Table 5B). Interestingly, two mTLE-HS patients clustered more with mTLE + HS patients (patients 20 and 28). We could not find any clinical parameters to explain this aberrant clustering (Table 1).

**Table 5:** Summary principle component analysis

**A**							
**Component-HC**							
**1**	**2**	**3**	**4**	**5**	**6**	**7**	**8**
**37.7%**	**13.4%**	**11.1%**	**7.5%**	**6.7%**	**5.3%**	**3.7%**	**3.2%**
IL-13	CCL4	CCL3	IL-6R	TIMP1	CCL19	TNFα	IL-23
IL-5	CCL2	IL-10	IL-18	IL-6	CCL5*	IL-1RA	CCL18
IL-22	IL-1α	ICAM1	Adiponectin	Cathepsin-S	IL-1β *	IL-4	
CCL19	IL-8	IL-27	IL-1β		MIF		
VEGF	CCL3	IL-25					
CCL22	CCL5	IL-21					
CXCL9		IFNα					
VCAM1		IL-6R					
IL-7							
HGF							
IL-23							
mTLE Δ	mTLE Δ	mTLE + HS Δ	ND	ND	mTLE + HS Δ	ND	ND
control	control	-HS & control			& control		
**B**							
**Component-CX**							
**1**	**2**	**3**	**4**	**5**	**6**	**7**	**8**
**40.1%**	**10%**	**8.4%**	**8%**	**5.8%**	**5%**	**4%**	**3.3%**
IL-5	IL-18	CCL2	Adiponectin	CXCL9	TIMP1	IL-1β	IL-25
VEGF	IL-6R	CCL4	ICAM1	CCL5	CXCL8.IL-8	IL-6	IL-21
VCAM1	MIF	Adiponectin	Cathepsin-S	HGF	IL-1α	IFNα	IL-4
CCL3	IL-1β	CXCL8.IL-8	CCL18		TNFα	IL-23	IL-10
CCL19	TNFα				IL-4		
IL-1RA							
IL-27							
IL-22							
IL-13							
IL-25							
CCL22							
IL-7							
CXCL9							
IL-1α							
IL-23							
TNFα							
mTLE Δ	ND	ND	ND	mTLE Δ	mTLE + HS Δ	ND	mTLE + HS Δ
control				control	& control		-HS & control

PCA identified 8 components in HC(A) and CX (B) that explain 88.5% (HC) and 84% (CX) of the variance in all the samples. The contribution of each component to the variance is given as percentage. Inflammatory mediators are listed in order of factor loading. Under each component list significant differences in this component between patient groups is indicated (delta). ND = no difference detected between component scores among the three patient groups.

Subsequently, we investigated possible correlations between the top three principle components of the hippocampal PCA analysis and several clinical parameters. Pearson’s correlation analyses revealed a significant correlation between HC component 3 and seizure frequency (including and excluding clustered seizures, see Table 1) in mTLE-HS patients. No correlation was found with the occurrence of spikes on the iEcOG. Interestingly, the expression levels of the two inflammatory mediators with the strongest loadings in HC component 3; CCL3 and IL-10, showed a correlation with seizure frequency (correlation coefficient of >0.7 with a *P* value below 0.05), however this correlation did not pass the strict Bonferroni correction for multiple testing.

In all, the data obtained from the PCA analyses showed that patient groups can be discriminated based on their expression profiles. The clustering in major components indicates that upregulation of inflammatory mediators in mTLE may involve multiple immunological pathways.

## Discussion

Over recent years, it has become apparent that the immune system plays a role in the development of mTLE [9,15,16]. The focus of protein studies on the immune pathology in mTLE thus far has mostly been on single cyto- and chemokines. However, experimental data from microarray studies [10,11] suggest that probably a whole network of cyto -and chemokines is activated in mTLE. Therefore, this study used a multiplex immunoassay (MIA) approach to measure multiple proteins of the immune system in the same mTLE samples.

We find a broad upregulation of inflammatory mediators in both HC and CX tissue of mTLE patients compared to autopsy controls. Up-regulated mediators include inflammatory proteins previously identified in mTLE, but also proteins not previously associated with mTLE. Network analysis showed that within patient groups there are two main protein networks with a high degree of co-regulation. Three components, obtained with principle component analyses on data of protein expression in the hippocampus, revealed that the three studied groups could be distinguished based on their expression profile.

Our data indicate that in human mTLE there are complex networks of upregulated inflammatory mediators, which may exert both pro- and anti-epileptogenic influences on the brain.

### Distinct expression patterns of upregulated inflammatory mediators

We measured 40 inflammatory proteins in the hippocampus and neocortex of autopsy control and mTLE patients. In mTLE tissue, 35 of these proteins were expressed above the detection limit. Sixty percent of these detectable mediators were significantly upregulated in mTLE tissue compared to autopsy controls, the remaining 40% showed no significant difference between the three patient groups (Table 2). We could distinguish two main patterns of upregulation in mTLE patients. Inflammatory mediators showing the first pattern (for example, IL-10 and IL-25) were upregulated in mTLE + HS patients (Figure 1A, 1B and 1G) compared to mTLE-HS and autopsy controls. Mediators showing the second pattern (for example, CCL4 and IL-7) were upregulated in both mTLE patient groups (+ and –HS) compared to controls (Figure 1C, 1D and 1G). Significantly more proteins (71.4%) showed the second pattern (upregulation in hippocampus and cortex) than the first (23.8%). Only in 19% of the patients upregulation of inflammatory mediators was confined to the hippocampus (Figure 1G, type A pattern). Thus, our results show that activation of inflammatory mediators is more widespread, and not restricted to the hippocampus, which often is the primary source of epileptic activity. Interestingly, IL1ra was upregulated only in the neocortex, but not in the hippocampus. The significance of the upregulation of this endogenous anti-inflammatory protein [46] remains to be determined. Upregulation may be a response of the cortex to seizure activity. Alternatively, the lack of IL1ra upregulation in the hippocampus may contribute to the epileptic properties of the hippocampus.

The upregulation of a specific subset of inflammatory mediators in the hippocampus of mTLE + HS patients is most likely associated with hippocampal sclerosis. These data indicate that the increased expression in mTLE patients is not just caused by post mortem changes in the autopsy control tissue. This latter point is further substantiated by the fact that covariate analysis in the controls revealed no effects of post mortem delay on any of the parameters measured. The human tissue samples used in our study are from patients suffering from repetitive seizures over a prolonged period. Therefore, part of the effects observed may be seizure-induced rather than specific for the mTLE pathogenesis. Moreover, we cannot rule out effects of antiepileptic drugs when comparing autopsy control patients with mTLE patients, although no correlations with any AED were found in our study. Further studies, particularly in animal models for TLE will be required to analyze the role of individual or groups of inflammatory mediators in epileptogenesis. Animal studies will also be essential to establish whether upregulation of specific inflammatory mediators have epileptogenic, or rather anti-epileptogenic, properties.

Our data show upregulation of protein levels of several inflammatory mediators, which previously have been found upregulated in human mTLE tissue. These include IL1β, CCL2, CCL3, CCL4, VCAM1, ICAM1, and VEGF [10,11,18,20,47,48], which were found to have pro-epileptogenic properties in animal models for TLE (for references: see Table 6). Particularly, IL-1β has frequently been associated with pro-epileptogenic properties, a protein which affects neuronal Ca^2+^ influx through NMDA-dependent signaling. As mentioned above, the endogenous antagonist of IL-1β, IL-1ra, has been attributed with seizure-inhibiting properties (Table 6).

**Table 6:** Summary of potential pro-and anti-epileptogenic properties of inflammatory mediators

**“Pro-epileptogenic”**			**“Anti-epileptogenic”**		
***Protein***	***Function in CNS***	***reference***	***Protein***	***Function in CNS***	***reference***
**CCL2**	- contributes to immune-cell recruitment across the BBB	[49]	**IL-1ra**	- An anti-infllammatory protein with seizure inhibiting properties in experimental TLE	[50-53]
**CCL3**	- Capable of inducing Ca^2+^ transients in neuronal and microglial cultures.	[22,54]	**IL-7**	Neurotrophic actions in embryonic brain cultures	[55,56]
	- Inhibition of systemic receptor leads to decrease in seizure activity				
**CCL4**	- Capable of inducing Ca^2+^ transients in neuronal and microglial cultures.	[22,54]	**IL-10**	- Inhibits development of seizures in FS and a hypoxia model for epilepsy.	[57-60]
				- SNPs that result in increased IL-10 are decreased in FS patients.	
	- Inhibition of systemic receptor leads to decrease in seizure activity				
				-Can give trophic support to neurons	
**CCL5**	- Capable of inducing Ca^2+^ transients in neuronal and microglial cultures	[22,49,54,61]	**IL-13**	- Protects BBB integrity.	[62]
	- Glut release from CNS cells in hypothalamus				
	- contributes to immune-cell recruitment across the BBB				
	- Inhibition of systemic receptor leads to decrease in seizure activity				
**CCL19**	immune-cell recruitment across the BBB.	[63,64]	**IL-25**	- Protects BBB integrity	[62,65]
**CCL22**	-Immune-cell recruitment across the BBB.	[66,67]	**IL-27**	Marked anti-inflammatory actions in EAE.	[68,69]
**CXCL9**	-Immune-cell recruitment across the BBB.	[70-72]	**IFNα**	- Suppression of hippocampal CA1 neurons & LTP	[73,74]
**IL-1α**	Polymorphisms that lead to increased transcription are associated with TLE	[75-77]	**HGF**	- Enhances neuronal survival	[78,79]
**IL-1β**	- Increased NMDA R subunit NR2B phosphorylation leading to increased Ca^2+^ influx	[80]^,^[25,52,81,82]	**VEGF**	- neuroprotective after experimental seizures	[83-85]
				- reduces hippocampal excitability	
	- Higher levels correlate with increased SRS after eFS				
**IL-5**	- Implicated in BBB disruption in CNS tumor bloodvessels	[86-88]	**IL-5**	- Anti-inflammatory actions in the periphery as a Th2 cytokine	[87-89]
	- Induces microglial proliferation				
				- Induces microglial activation	
**IL-7**	Induces neuronal apoptosis in human NT2 cells	[90]			
**IL-22**	- BBB disruption through down-regulation of occludin.	[91]			
**VEGF**	BBB disruption → reduction in tight junction proteins in kainate model for TLE	[92,93].			
**ICAM-1**	- implicated in BBB disruption	[20]			
**VCAM-1**	- Immune-cell recruitment across the BBB	[20]			

Abbreviations: *BBB* blood brain barrier, *CNS* central nervous system, *SRS* spontaneous recurrent seizure activity, *NMDA N*-methyl *D*-aspartate, *NR2B* N-methyl D-aspartate receptor subtype 2B, *eFS* experimental febrile seizures, *NT2* neuronal teratocarcinoma Tera 2, *FS* febrile seizures, *LTP* long term potentiation, *EAE* experimental autoimmune encephalitis, *SNP* single nucleotide polymorphism.

Two proteins identified in this study have been described in human genetic studies before: single nucleotide polymorphisms (SNPs) present in IL-1α and IL-10 genes are associated with TLE and febrile seizures respectively (Table 6).

Our data showing upregulation of these pro-epileptogenic proteins in mTLE confirm that these genes are regulated, and that upregulation of transcripts translates into increased production of protein in mTLE tissue.

In this study, we identified a series of new inflammatory mediators that show increased protein levels in human mTLE tissue. These include CCL5, CCL19, CCL22, CXCL9, IL-5, IL-7, IL-13, IL-22, IL-25, IL-27, IFNα and HGF. As the majority of these new mTLE-associated inflammatory mediators have not been studied in animal models of TLE, information on pro- or anti-epileptogenic properties is limited. However, most of these proteins have previously been studied in relationship to other CNS pathologies. Processes such as neuroprotection, excitotoxicity through Ca^2+^ influx, glutamate/GABA signaling and disruption of the BBB have been studied. These processes potentially can influence epileptogenesis [94-97]. Based on these studies, we categorized the newly identified inflammatory mediators in mTLE as potentially pro-epileptogenic or anti-epileptogenic (Table 6). Potentially pro-epileptogenic are all chemokines, the interleukins IL-5, 7 and 22, and the adhesion molecules VCAM1 and ICAM1. Potentially anti-epileptogenic are IL-1ra, IL-10, 13 and 25, IFNα and HGF. Studies on the potential role of IL-5, IL-7 and VEGF in processes related to epileptogenesis provide inconsistent information. [47,98].

IL-6 or TNFα protein levels did not differ between the three patient groups. Even though both cytokines have been implicated in epilepsy before [8,9,15,99], as we did not repeat the experiment with multiple antibodies, we cannot rule out technical issues. Our results however, are in line with data from mRNA profiling studies on human TLE tissue [10,11,14,15,100] as in these studies no significant differences in mRNA or protein were detected in brain tissue. Increased levels of IL-6, but not of TNFα, were found in plasma or cerebrospinal fluid (CSF) of TLE patients [101,102]. Therefore, increased IL-6 levels might not arise from brain tissue [103-105] but more likely from peripheral blood mononuclear cells (PMBCs). Indeed, upon stimulation, PMBCs from epileptic patients released increased amounts of IL-6 compared to controls [106].

Thus, our total set of upregulated inflammatory mediators comprises (potentially) pro- as well as anti-epileptogenic proteins in both mTLE patient groups.

Interestingly, we detected both CCL4 and IL-25 in glial cells surrounding blood vessels (Figures 4 and 5). Here, these proteins probably exert opposing influences on epileptogenesis, as CCL4 is thought to disrupt the blood–brain barrier (BBB) and attract immune cells across the BBB while IL-25 has been proposed to have a protective effect on the BBB (Table 6).

### Correlation and principle component analyses reveal complex immune networks in mTLE

To study co-regulation of inflammatory mediators, we first performed bivariate correlation network analysis on the datasets within each patient group. This analysis revealed co-regulation of inflammatory mediators in one large and one small network in each patient group (Figure 6). We observed that in most cases, patients with high levels of the smaller network components (for example, CCL2, CCL4, IL-8) had lower expression levels of larger network components (for example, IL-13, IL-5 CCL9) and vice versa. Subsequent PCAs on all investigated brain samples confirmed these networks and revealed eight components of clustered inflammatory proteins in both the HC and the CX separately (Figure 7, Table 5A and B). Each component typically contained both putative pro- and anti-epileptogenic proteins, suggesting that these proteins are co-regulated in all patient groups. We detected the majority of mTLE-specific differences in the HC of mTLE patients. Further analysis of the HC components shows that individual patients segregate into the three patient groups (Figure 7A), indicating that each patient group has a unique immune profile. The first two components, which explained the greatest variance of protein expression in the HC, contained only proteins of a type II pattern (that is, upregulated in both mTLE patient groups), whereas the majority of component 3 consists of proteins with a type I expression pattern, and this is clearly shown in the PCA plots (Figure 7A). As expected, the majority of HC component 1 consisted of proteins also detected in the large network detected in the bivariate correlation network analysis. HC component 2 comprises chemokines represented in the smaller correlation network (Figure 6).

The PCA analysis on the data set from the CX tissue also showed segregation of patients into patient groups (Figure 7B), although less pronounced, possibly due to the smaller number of regulated proteins. As expected, there was substantial overlap between the major clusters found in the HC and CX, as nine of the ten inflammatory mediators clustered in the HC component 1 were also present in the CX component 1 (Figure 7, Table 5).

The data obtained from the PCAs suggest that the activated immune system in mTLE is complex and may comprise different networks and pathways. The biological function and pathological outcome of activation of the immunological networks needs to be further investigated. Surprisingly, pro- and anti-epileptogenic inflammatory mediators cluster together, suggesting co-regulation of these two types of responses. Correlation analysis of upregulated whole networks with clinical parameters revealed a significant correlation between the HC component 3 and seizure frequency in the hippocampus of mTLE-HS patients. Indeed, the top two proteins in this component (CCL3 and IL-10) showed a weak correlation with seizure frequency in the hippocampi of mTLE-HS patients only. No correlations were found with iEcOG spikes. Larger patient populations will be required to further elucidate the relevance of upregulation of inflammatory networks or specific inflammatory mediators for patient pathology.

In summary, we observed a widespread upregulation of inflammatory mediators in the mTLE brain. Co-regulation of upregulated mediators indicates that they cluster in complex immunological networks. Within these networks we identified both pro- and anti-inflammatory mediators. Detailed analysis of these complex pathways in animal models will be required to assess their putative role in epileptogenesis. Our data suggests that the success of any future therapeutic strategies to treat mTLE will require a multifactorial approach aimed at blocking detrimental effects of proinflammatory pathways while promoting endogenous anti-inflammatory pathways. Promising targets for treatment would be, for instance, CCR5 receptors, which can be activated by a number of chemokines [54]. CCR5 inhibitors are currently being tested in clinical trials for HIV/AIDS treatment [107,108]. Treatment with CCR5 antagonists will inhibit the action of pro-epileptogenic chemokines CCL3, 4 and 5, the major chemokines in the second immunological network we identified in the HC. Inhibition of CCL3, 4 and 5 signaling, and in general proinflammatory pathways, may contribute to tipping the balance from a perturbed pro-epileptogenic to a more protective and anti-epileptogenic immune system in mTLE patients.

## Competing interests

The authors certify that they have no competing financial interests.

## Authors’ contributions

The work presented here was carried out in collaboration between all authors. AK, ON and PG defined the research theme and designed the methods and experiments. AK and MW gathered and prepared the human tissues for the experiments. WJ carried out the multiplex ELISA and discussed and aided with the interpretation. AK performed the IHC and IFC. CH and MZ aided with the PCA analysis and the interpretation of the networks. PR and PG were the neurosurgeons responsible for the removal of brain tissue, CF carried out the intraoperative electrocorticography. AK drafted the manuscript and in conjunction with ON and PG presented this paper. All authors have read and approved the final version of the manuscript.
